# PK/PD Analysis by Nonlinear Mixed-Effects Modeling of a Marbofloxacin Dose Regimen for Treatment of Goat Mastitis Produced by Coagulase-Negative Staphylococci

**DOI:** 10.3390/ani11113098

**Published:** 2021-10-29

**Authors:** Augusto Matías Lorenzutti, Juan Pablo Vico, Juan Manuel Serrano-Rodríguez, Martín Alejandro Himelfarb, Manuel Ignacio San Andrés-Larrea, José Julio de Lucas-Burneo, Nicolás Javier Litterio

**Affiliations:** 1 Facultad de Ciencias Agropecuarias, IRNASUS CONICET-Universidad Católica de Córdoba, Córdoba X5016DHK, Argentina; matiaslorenzutti@ucc.edu.ar (A.M.L.); juanpablo.vico@ucc.edu.ar (J.P.V.); martinhimelfarb@ucc.edu.ar (M.A.H.); nlitterio@ucc.edu.ar (N.J.L.); 2 Department of Nursing, Pharmacology and Physiotherapy, Pharmacology Area, Faculty of Veterinary Medicine, Universidad de Córdoba, 14071 Córdoba, Spain; 3 Department of Pharmacology and Toxicology, Faculty of Veterinary Medicine, Universidad Complutense de Madrid, 28040 Madrid, Spain; misanand@vet.ucm.es (M.I.S.A.-L.); delucas@vet.ucm.es (J.J.d.L.-B.)

**Keywords:** marbofloxacin, pharmacokinetic, pharmacodynamic, mastitis, goats, coagulase negative staphylococci

## Abstract

**Simple Summary:**

Coagulase-negative staphylococci are main pathogens that produce goat mastitis. Marbofloxacin is a third-generation fluoroquinolone approved to treat mastitis in animals. Since the efficacy of an antimicrobial is related with its concentration in the site of infection, and the latter depends of dose and biological processes that determine the distribution of the antimicrobial in different tissues and secretions, the objectives of this study were to evaluate the efficacy of a dose regimen of marbofloxacin (10 mg/kg/24 h) administered intramuscularly for five days in goats with mastitis induced by coagulase-negative staphylococci, by an evaluation of the concentrations of marbofloxacin achieved in blood and milk over time (called pharmacokinetics), and characterizing the concentration–effect relationship of marbofloxacin against coagulase-negative staphylococci in Mueller Hinton broth and goat milk, by time kill assays, in order to determine the concentrations of marbofloxacin related with an adequate bacterial count reduction (measured by efficacy index AUC/MIC). The proposed dose regimen was adequate for the treatment of goat mastitis produced by coagulase-negative staphylococci, resulting in a microbiological and clinical cure of all animals. The animal model used in this study provided important pharmacokinetic information about the effect of the infection on the pharmacokinetics of marbofloxacin. Pharmacodynamic modeling showed that marbofloxacin concentrations needed for antimicrobial efficacy were higher in goat milk compared with Mueller Hinton broth. Bacterial resistance to antimicrobials is a serious problem, since marbofloxacin is considered a critically important antimicrobial, and its rational and prudent use could extend its utility over time.

**Abstract:**

Coagulase-negative staphylococci are main pathogens that produce goat mastitis. Marbofloxacin is a third-generation fluoroquinolone approved for treat mastitis in animals. The objectives of this study were: (i) to determine the pharmacokinetics of marbofloxacin (10 mg/kg/24 h) in serum and milk administered intramuscularly for five days in goats with mastitis induced by coagulase-negative staphylococci; (ii) to characterize the concentration–effect relationship of marbofloxacin against coagulase-negative staphylococci in Mueller Hinton broth and goat milk; (iii) to determine AUC/MIC cutoff values of marbofloxacin, and (iv) to perform a PK/PD analysis to evaluate the efficacy of the dose regimen for the treatment of goat mastitis produced by coagulase-negative staphylococci. Marbofloxacin presented context-sensitive pharmacokinetics, influenced by the evolution of the disease, which decreased marbofloxacin disposition in serum and milk. Marbofloxacin showed a median (95% CI) *f*AUC/MIC values for MIC of 0.4 and 0.8 µg/mL of 26.66 (22.26–36.64) and 32.28 (26.57–48.35) related with −2 log_10_CFU/mL reduction; and 32.26 (24.81–81.50) and 41.39 (29.38–128.01) for −3 log_10_CFU/mL reduction in Mueller Hinton broth. For milk, −2 log_10_CFU/mL reduction was achieved with 41.48 (35.29–58.73) and 51.91 (39.09–131.63), and −3 log_10_CFU/mL reduction with 51.04 (41.6–82.1) and 65.65 (46.68–210.16). The proposed dose regimen was adequate for the treatment of goat mastitis produced by coagulase-negative staphylococci, resulting in microbiological and clinical cure of all animals. The animal model used in this study provided important pharmacokinetic information about the effect of the infection on the pharmacokinetics of marbofloxacin. Pharmacodynamic modeling showed that *f*AUC/MIC cutoff values were higher in goat milk compared with Mueller Hinton broth.

## 1. Introduction

Mastitis is one of the most important infectious pathologies in dairy goats, producing economical losses, and affecting health and animal welfare. Coagulase-negative staphylococci (CNS) are the main microorganisms that produce subclinical and clinical mastitis in this species, with a prevalence that could be higher than 71% [1,2].

The use of a pharmacokinetic/pharmacodynamic (PK/PD) approach is the main tool to study the relationship between the disposition and exposition of an antimicrobial to a microorganism and its effect on the bacterial population, and could help in the design of optimal dose regimens that result in maximal efficacy, minimizing the emergence of antimicrobial resistance and toxicity [3,4,5,6,7].

Marbofloxacin (MFX) is a third generation fluoroquinolone approved for veterinary use, and is indicated for pyoderma, otitis, digestive, respiratory, urinary and reproductive tract infections, such as endometritis, pyometra or mastitis [8].

Pharmacokinetics of marbofloxacin in lactating and non-lactating goats have been described in several studies [9,10,11,12,13,14,15]. However, to the authors knowledge, only two studies evaluated pharmacokinetics and milk penetration of marbofloxacin in lactating goats [16,17]. In fact, in these studies, MFX presented a good penetration to goat milk. Moreover, a PK/PD analysis by Monte Carlo simulation was carried out by Lorenzutti et al. (2017) [16] using single-dose pharmacokinetic data obtained from healthy lactating goats, and then, a multi-dose simulation was made in order to estimate the steady-state C_max_ and AUC_24_ of MFX. Additionally, MIC and MPC of MFX were determined from 106 regional CNS strains isolated from goat mastitis. This study has concluded that an optimal dose regimen of approximately 10 mg/kg/24 h could be used in order to achieve a good antimicrobial efficacy against goat pathogens as CNS or *Mycoplasma agalactiae*.

The pharmacokinetic information derived from studies using healthy individuals could be interpreted carefully. More precisely, the pharmacokinetics of gatifloxacin was significantly affected by the presence of mastitis in goats. In this study, gatifloxacin presented a higher penetration and permanence in milk of goats with clinical mastitis [18]. In this manner, pharmacokinetic data obtained from animal models with disease should be encouraged in order to conduct more realistic PK/PD analysis.

Nonlinear mixed-effects modeling is an alternative approach to the traditional pharmacokinetic analysis, and the main advantages that present are a robust estimation of population parameters and the inter-individual variability (IIV). Moreover, this approach can determine the effect of different covariates on the variability of the parameters, therefore, simulations derived from this method allow us to increase the sample size and include multiple scenarios, that could be difficult to conduct in experimental studies. For these reasons, nonlinear mixed-effects models are a better option to perform PK/PD analysis with antimicrobials [19,20,21].

In the study of Lorenzutti et al. (2017) [16], the pharmacodynamic parameter used to conduct the PK/PD analysis was the MIC value of MFX against regional CNS isolated from goats with mastitis. MIC is a static pharmacodynamic parameter that presents many limitations. As an alternative, in vitro static or dynamic time kill curves (TKC) studies and pharmacodynamic modelling can provide more detailed information about the concentration–effect relationship, describing the time course of the antimicrobial effect [22]. TKC experiments are one of the most accurate ways to determine the cutoff values for PK/PD indexes. Cutoff values of a PK/PD index could be affected by different factors such as the microorganism, individual factors (immune system) or the culture medium. Most of PK/PD endpoints are determined in serum or broth, but in the case of mastitis, it could be necessary to determine the PK/PD endpoints for milk.

In this context, the main objectives of this study were: (i) to determine the pharmacokinetic behavior of MFX (10 mg/kg/24 h) in serum and milk, administered by IM route in a multi-dose regimen of five days in goats with mastitis induced by CNS, by nonlinear mixed-effects analysis; (ii) to characterize the concentration-effect relationship of MFX against CNS in Mueller Hinton broth (MHB) and goat milk by nonlinear mixed-effects analysis; (iii) to determine the population *f*AUC/MIC cutoff values of MFX in MHB and goat milk, and (iv) to perform a PK/PD analysis to evaluate the efficacy of the MFX dose regimen for the treatment of goat with mastitis produced by CNS.

## 2. Materials and Methods

### 2.1. Animals, Treatments and Samples

This research was approved by the Commission of Bioethics and Animal Welfare of the Faculty of Agricultural Sciences of Catholic University of Córdoba (CBBA.08.2014.UCC). To conduct the pharmacokinetic study of a multi-dose regimen of MFX by IM route, seven female, Anglo Nubian, non-pregnant lactating goats with sub-clinical mastitis were enrolled. The animals weighed 39.46 ± 5.43 kg and aged 3.79 ± 0.76 years. Total milk production was 0.38 ± 0.23 L/day. The selection process of animals with subclinical mastitis was carried out by the following inclusion criteria: initially, a physical examination of both udders of each animal was performed, as well as the macroscopic characteristics of the milk, in order to identify those animals with clinical mastitis and exclude them from the study. In parallel, the milk from each mammary gland was analyzed using the California mastitis agglutination test (California mastitis test, CMT). Individuals with CMT results ≥ 2 were pre-selected. Subsequently, milk samples were taken from each mammary gland suspected of subclinical mastitis, and microbiological culture and somatic cell count were performed in order to diagnose subclinical mastitis. From the pool of goats with subclinical mastitis produced by CNS, only those that presented one udder infected were enrolled, using the other mammary gland as a control. Animals did not receive any medication one month previous to the beginning of the experience. Goats had free access to water and alfalfa bales during the experiments. Previous to the beginning of the study, milk production, pH and somatic cell count of each mammary gland were recorded (Table 1).

The pharmacokinetic study was carried out according the optimal MFX dose calculated by Lorenzutti et al. (2017) [16]. The multi-dose regimen was conducted by administration of MFX (10 mg/kg/24 h) by IM route for five days. Serum and milk samples were taken before MFX administration. After MFX administration of 10 mg/kg/24 h by IM route, blood samples were taken at 10, 20, 30 and 45 min, and 1, 1.5, 2, 4, 6, 8, 10, 12 and 24 h for the first, third and fifth administrations, and at 30 and 45 min and at 1, 2, 10 and 24 h for the second and fourth administrations. Milk samples of 10 mL from each mammary gland were taken at 1, 2, 4, 6, 8, 10, 12 and 24 h for the first, third and fifth administrations and at 1, 2, 10 and 24 h for the second and fourth administrations. Goats were milked every 24 h, and milk production, SCC and pH were recorded. Before milking, samples for microbiological culture were taken from each mammary gland daily. Samples for microbiological evaluation were taken according to aseptic methods, recommended by the National Mastitis Council [23]. For this, sterile plastic tubes were used. Each teat was first disinfected with a cotton swab soaked in 70% alcohol and dried with individual paper towels to avoid cross contamination. The first 3 milk jets were discarded to eliminate pathogens present in the teat canal, and 5 to 15 mL of milk were collected, refrigerated at 4 °C and immediately transported to the laboratory.

### 2.2. Marbofloxacin Determination

Serum and milk concentrations of MFX were determined by high-performance liquid chromatography with ultraviolet detector (HPLC/uv), which is a modification of the method reported by Waxman et al. (2001) [9]. MFX was provided by Fluka Aldrich Sigma and ofloxacin (used as internal standard) by Sigma Chemical. HPLC determinations were prepared under the following chromatographic conditions: Kromasil 100 C18 5 μm 150 × 4.6 mm column and a Kromasil C18 5 μm 30 × 4.6 mm guard column, both operated at room temperature. The mobile phase consisted of buffer pH 2.7–methanol–acetonitrile–acetic acid trimethylamine (74:20:4:1:1). The buffer pH 2.7 was a 0.4% aqueous solution of tetrabutylammonium hydrogen sulphate and diammonium hydrogen phosphate. The UV detection wavelength was 295 nm, and the flow rate was 0.6 mL/min.

For HPLC validation, linear calibration curves were calculated for low (0.025–0.5 μg/mL; *R*2: 0.9961) and high (0.5–15 μg/mL; *R*2: 0.9969) concentration range for serum, and low (0.025–0.5 μg/mL; *R*2: 0.9985) and high (0.5–10 μg/mL; *R*2: 0.9976) concentration range for milk. The lower limit of quantification (LLOQ) for MFX in serum and milk was 0.025 μg/mL. The precision of LLOQ was 5.95% and 5.59% in serum and milk, respectively. Accuracy of LLOQ was 84.61% ± 0.29% and 89.06% ± 0.34% for serum and milk, respectively. The intra-assay reproducibility for serum and milk was 4.14% ± 1.83% and 3.85% ± 1.85%, respectively. The inter-assay reproducibility for serum and milk was 6.70% ± 3.95 and 8.11% ± 5.22%, respectively.

### 2.3. Pharmacokinetic Modeling and Simulation

**Figure 1:**
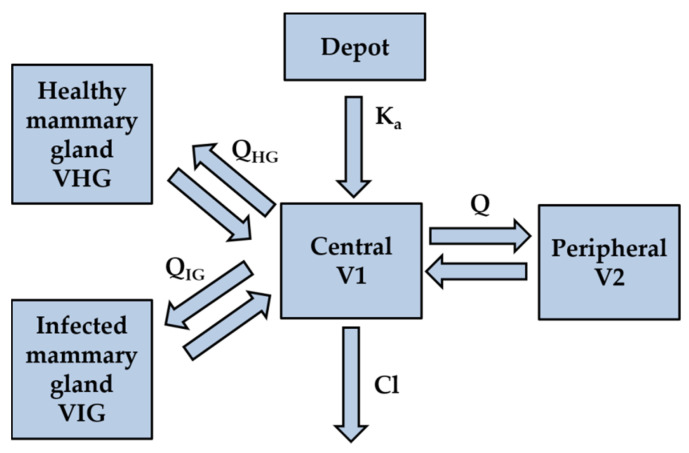
Schematic diagram of the modified bi-compartmental model of MFX, administered by IM route in goats with mastitis.

Pharmacokinetic data of free MFX concentrations in serum and milk were analyzed by nonlinear mixed-effects models using a modified bi-compartmental model including two extra compartments representing each mammary gland, with a proportional error model, with Monolix Suite 2020R1 software (Lixoft, Antony, France). A schematic diagram of the model is presented in Figure 1.

The pharmacokinetic parameters estimated by the model were: K_a_ (absorption constant), Cl (clearance of the central compartment), V1 (volume of distribution of the central compartment), Q (inter-compartmental clearance between central and peripheral compartments), V2 (volume of distribution of the peripheral compartment), Q_HG_ (inter-compartmental clearance between central and healthy mammary gland compartments), VHG (volume of distribution of the healthy mammary gland), Q_IG_ (inter-compartmental clearance between central and infected mammary gland compartments), and VIG (volume of distribution of the infected mammary gland compartment). A log-normal distribution was assumed for all pharmacokinetic parameters of the model.

Since multiple administrations of MFX were included in the study, each administration interval (24 h) was considered as a different occasion in the model. Additionally, an emptying effect was established in each mammary gland compartment in order to include the milking effect of the udder every 24 h [24].

The covariates weight, age, total milk production (pool of both mammary glands), milk production of healthy mammary gland, milk production of infected mammary gland and the day of treatment (as categorical covariate) were evaluated in order to determine the possible effects of these covariates on pharmacokinetic parameters of MFX. A covariate was included in the final model if presented statistical significance (*p* ˂ 0.05), and reduced the IIV and the likelihood ratio tests (LRT) as −2·log-likelihood (−2LL), Akaike information criterion (AIC), and Bayesian information criterion (BIC) [24,25].

The final covariate model can be expressed in a general form:

$$\mathrm{Individual}\;\mathrm{parameter}={\;\theta }_{\mathrm{POP}}\cdot e^{\beta \mathrm{COV}\theta }\cdot e^{\eta \theta }$$

where θpop is the population parameter estimate, βcovθ is the covariate parameter, and ηθ is the IIV.

The proportional error model is represented in the general form:

$${\mathrm{CONC}}_{\mathrm{CC}}={\;C}_{C}+b_{1}\cdot {\;C}_{C}\cdot e$$

$${\mathrm{CONC}}_{\mathrm{HG}}={\;C}_{\mathrm{HG}}+b_{2}\cdot {\;C}_{\mathrm{HG}}\cdot e$$

$${\mathrm{CONC}}_{\mathrm{IG}}={\;C}_{\mathrm{IG}}+b_{3}\cdot {\;C}_{\mathrm{IG}}\cdot e$$

where CONC_CC_, CONC_HG_ and CONC_IG_ are the observed concentration, and C_C_, C_HG_ and C_IG_ are the predicted concentrations of the central, healthy mammary gland and infected mammary gland compartments, respectively.

Based on the final model, 1000 pharmacokinetic profiles of the multi-dose regimen of MFX (10 mg/kg/24 h) by IM route for five days were simulated using Simulx 2020R1 software (Lixoft, Antony, France). The MFX mean free-AUC values (*f*AUC) of the five administrations were calculated in serum and milk of the infected mammary gland for each simulated subject. Then, 95% confidence interval (95% CI) of *f*AUC/MIC for MIC values of 0.4 and 0.8 µg/mL were calculated and used for PK/PD analysis.

### 2.4. Time Kill Curves Assays

A static pharmacodynamic assay was conducted in order to characterize the concentration–effect relationship of MFX against CNS strains. For this purpose, thirteen CNS strains isolated from goats with mastitis previously used in the study of Lorenzutti et al. (2017) [16] were selected. MIC values of each strain were previously determined by microdilution method [26], and only strains with MIC values of 0.4 µg/mL (corresponding to MIC_90_) and 0.8 µg/mL were included. After overnight incubation in Mueller Hinton agar plates at 37 °C, CNS strains were suspended in 10 mL of normal saline. Bacterial suspensions were further diluted in test tubes to achieve a final inoculum close to 5 × 10^7^ CFU/mL [26]. One mL of the bacterial suspension was added to 9 mL of cation-adjusted Mueller Hinton broth (CAMHB) or goat milk, and then, MFX was supplemented to each tube in order to achieve a final concentration from 0, 0.5, 1, 2, 4 and 8 MIC. Tubes were incubated at 37 °C during 24 h and aliquots of 100 µL were taken at 0, 2, 5, 8, 12 and 24 h and serial ten-fold dilutions were made for bacterial count in Mueller Hinton agar plates.

It is known that in vitro pH of milk with mastitis pathogens can decrease between 1–1.5 fold [27,28]. Moreover, the acidity can reduce the antibacterial activity of fluoroquinolones as reported in other study [29]. For this reason, pH in milk was measured previously in another assay. Values close to 5.8 and 5.2 after 24 h for low and high inoculum were obtained. Therefore, milk was buffered with HEPES at 100 mM [27].

Before pharmacodynamic data analysis, drug concentrations in each medium were transformed into free concentrations using the unbound fractions in plasma and goat milk previously determined in our laboratory with values of 0.71 in milk, and 0.73 in plasma [17]. On the other hand, it was also determined in CAMHB with a value close to 0.923.

### 2.5. Pharmacodynamic Modeling and Simulation

**Figure 2:**
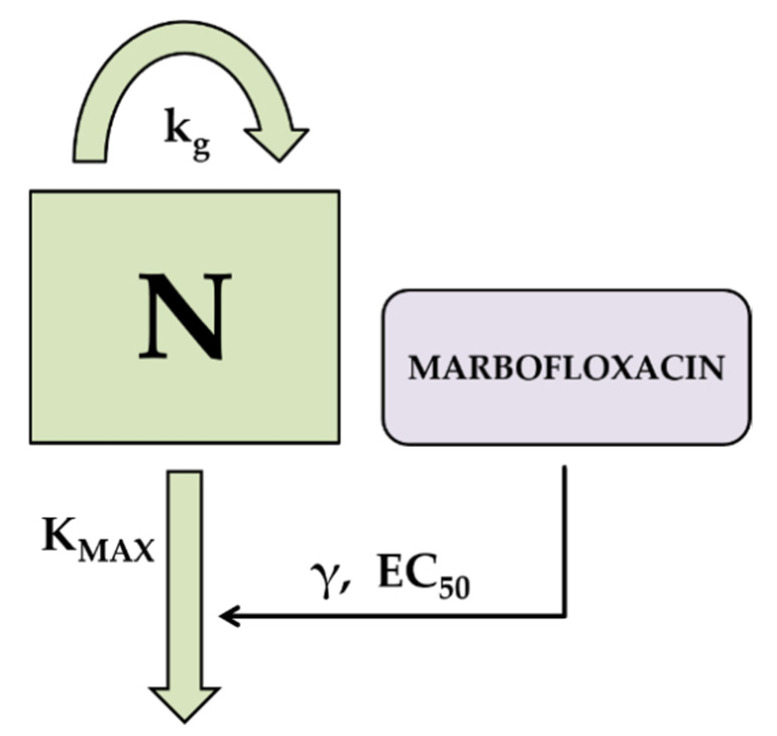
Schematic diagram of the pharmacodynamic model used for time kill curves of MFX against CNS.

Time kill curves data were analyzed by nonlinear mixed-effect models using Monolix Suite 2020R1 software (Lixoft, Antony, France). The semi-mechanistic model used was previously described [22,30] and consisted in a bacterial sub-model characterizing the logistic growth of the bacterial population, and a PK/PD model characterizing the antimicrobial drug effect. The application of these models allow to describe the evolution of bacterial counts over time, when bacterial population is exposed to different concentrations of an antimicrobial. A schematic diagram of the semi-mechanistic model is presented in Figure 2, and can be represented by the following equation:

$$\frac{\mathrm{dN}}{\mathrm{dt}}=k_{g}\cdot \left(1-\frac{N}{N_{\max}}\;\right)\cdot N-K_{\max}\cdot \frac{C^{\gamma }}{C^{\gamma }+{\mathrm{EC}}_{50}^{\gamma }}\;\cdot N$$

where N is the bacterial count, expressed as log_10_ CFU/mL; k_g_ is the net growth rate of the bacterial population, N_max_ is the maximum bacterial count at stationary phase; K_max_ is the maximum killing rate; EC_50_ is the concentration that produce 50% of the maximum effect and γ is the sigmoidicity factor.

It was assumed that all model parameters were log-normally distributed, and a constant error model was that best fitted the data and was used in the final model, represented by the following equation:

CFU= N+a·e

where CFU is the observed bacterial count and N is the predicted bacterial count, respectively.

The culture medium (CAMHB or milk) and the MIC value of each CNS strain were included in the analysis as covariates, in order to evaluate its effects in the parameters of the model. Covariates were included in the final model if presented statistical significance (*p* ˂ 0.05), and reduced the IIV and the likelihood ratio tests (LRT) as −2·log-likelihood (−2LL), Akaike information criterion (AIC), and Bayesian information criterion (BIC) [25].

Based on the final pharmacodynamic model of MFX against CNS strains in CAMHB and milk, a simulation of 2000-time kill curves (1000 for each medium) were performed with Simulx 2020R1 software (Lixoft, Antony, France). The original free MFX concentrations used in the static pharmacodynamic assay were included in the simulation.

### 2.6. Determination of the PK/PD Cutoff Values

From the results of time kill curves simulations, an I_max_ model with a proportional error model was used to characterize the relationship between MFX exposition (*f*AUC/MIC values) and the difference of bacterial count between 0 and 24 h, with Monolix Suite 2020R1 software (Lixoft, Antony, France). The structural model was:

$$E=E_{0}-I_{\max}\cdot \frac{C^{\gamma }}{C^{\gamma }+{\;\mathrm{IC}}_{50}^{\gamma }}$$

where E is the 0–24 h bacterial count difference (log_10_ CFU/mL) at a certain *f*AUC/MIC value, E_0_ is the bacterial count difference at *f*AUC/MIC = 0, I_max_ is the maximum reduction in bacterial count difference, *C* is the *f*AUC/MIC value, IC_50_ is the *f*AUC/MIC value that produce 50% of I_max_ and γ is the sigmoidicity factor.

The culture medium (CAMHB or milk) and the MIC value of the CNS strains were included in the analysis as covariates, in order to evaluate its effects in the parameters of the model. Covariates were included in the final model if they presented statistical significance (*p* ˂ 0.05), and reduced the IIV and the likelihood ratio tests (LRT) as −2·log-likelihood (−2LL), Akaike information criterion (AIC), and Bayesian information criterion (BIC) [25].

The final model was used to simulate 1000 exposure–count difference curves with Simulx 2020R1 software (Lixoft, Antony, France). The entire range of *f*AUC/MIC exposition of MFX used in the original experiment was included in the simulation. Simulated data were used to determine the *f*AUC/MIC cutoff values related with reductions in −1, −2 and −3 log_10_ CFU/mL.

### 2.7. PK/PD Analysis of Marbofloxacin against Coagulase Negative Staphylococci Isolated from Goat Mastitis

Pharmacokinetic and pharmacodynamic data obtained from simulations were used to conduct a PK/PD analysis in order to determine the antimicrobial efficacy of the proposed multi-dose regimen of MFX for mastitis produced by CNS in goats.

Marbofloxacin *f*AUC/MIC values corresponding to the first administration (*f*AUC_first_/MIC) and the mean AUC value of five administrations (*f*AUC_mean_/MIC), in serum and milk of infected mammary glands, were included in the I_max_ model, and 1000 exposition-count difference curves in serum and milk (500 for each MIC value) were simulated with Simulx 2020R1 software (Lixoft, Antony, France), and 95% CI of log_10_ CFU/mL differences were recorded in order to determine the antimicrobial efficacy of the proposed dose regimen of MFX taking into account the pharmacokinetic and pharmacodynamic IIV.

Finally, PK/PD results were contrasted with the evolution over time of milk production, SCC and pH, as well as microbiological cure data obtained from microbiological culture of infected mammary glands.

### 2.8. Statistical Analysis

Milk production, SCC and pH data from healthy and infected mammary glands were analyzed by ANOVA for paired samples test. Differences in culture medium, MIC and log_10_CFU/mL reductions for *f*AUC/MIC data, and differences in milk production between healthy and infected mammary glands during marbofloxacin treatment were analyzed by a generalized linear mixed-effects model, using a Gamma family and “log” link function. All tests were conducted using Infostat^®^ 2018 (Grupo InfoStat, FCA, Universidad Nacional de Córdoba) software.

## 3. Results

### 3.1. Pharmacokinetic Modeling and Simulation

All animals finished this study with no clinical evidence of adverse/toxic effects after administration of MFX. No irritation, inflammation or swelling were observed in the site of administration during the experience.

**Figure 3:**
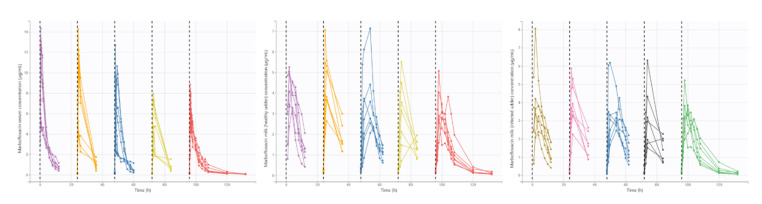
Serum and milk (healthy and infected mammary glands) free concentrations of MFX administered at a dose of 10 mg/kg/24 h for five days in goats with mastitis produced by CNS (*n* = 7). Different colors indicate different administrations. Dashed black lines indicate MFX administration.

**Figure 4:**
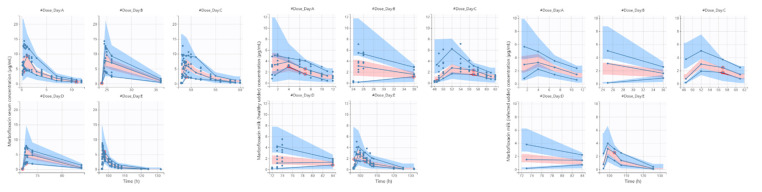
Visual predictive check for serum and milk (healthy and infected mammary glands) free concentrations of MFX administered at a dose of 10 mg/kg/24 h for five days in goats with mastitis produced by CNS (*n* = 7).

Pharmacokinetic profiles of MFX after IM administration of 10 mg/kg/24 h during five days in serum and milk of each mammary gland (healthy and infected) are presented in Figure 3. After the pharmacokinetic analysis by nonlinear mixed-effects models (Table 2), K_a_, Cl, Q, Q_HG_ and VHG presented significant differences among the different doses of MFX. Respecting day 1, K_a_ increased in day 2 and then decreased over time; Cl trend to decrease among successive administrations; Q was significantly lower at days 2 and 3; and finally Q_HG_ and VHG were lower at days 4 and 5. Moreover, the volume of healthy mammary gland significantly increased V1. The final model showed a good capability to predict serum and milk concentrations of MFX, with most observed values falling into 95% prediction intervals (Figure 4). Plots of predicted versus observed concentrations, population/individual-weighted residuals (PWRES and IWRES) versus predictions/time, showed residuals uniformly distributed around the predictive values.

**Figure 5:**
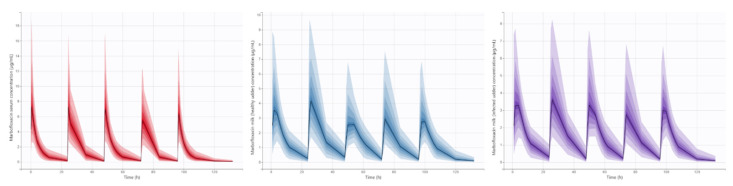
The 95% CI of simulated serum and milk (healthy and infected mammary gland) free concentrations of MFX administered at a dose of 10 mg/kg/24 h for five days in goats with mastitis produced by CNS (*n* = 7).

The final pharmacokinetic model was exported to Simulx, a simulation software of Monolix Suite. The same MFX dose regimen of 10 mg/kg/24 h by IM route was replicated, and a simulation of 1000 individuals was carried out in order to obtain serum and milk (from healthy and infected mammary glands) concentration-time profiles of MFX (Figure 5). Additionally, *f*AUC_first_/MIC and *f*AUC_mean_/MIC values for each simulated individual were determined for serum, and milk of the infected mammary gland. The 95% CI of *f*AUC_first_/MIC and *f*AUC_mean_/MIC of MFX for serum and milk of the infected mammary gland are presented in Table 3.

### 3.2. Pharmacodynamic Modeling and Simulation

**Figure 6:**
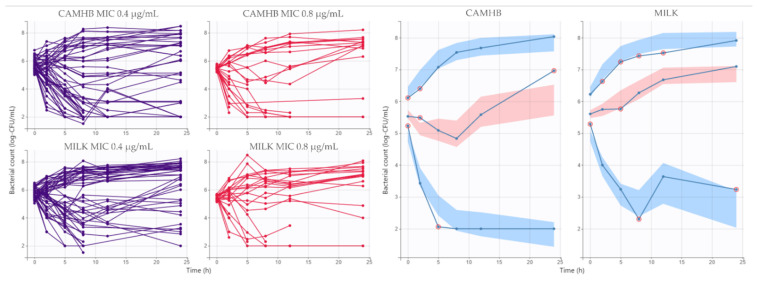
Bacterial count profiles of CNS for different expositions to MFX and visual predictive check of the final model.

Time kill experiments were conducted in order to characterize the concentration-effect relationship of MFX against CNS isolated from goat mastitis, and a static PK/PD model was selected using nonlinear mixed-effects models. Bacterial count over time and visual predictive check of the final model for CAMHB and goat milk are presented in Figure 6.

In the final model, goat milk used as medium culture significantly increased EC_50_, and MIC decreased N_0_ and increased EC_50_ (Table 4). Correlation between k_g_ and K_max_ were included in the final model. The model fitted the data well, with most of the observed bacterial count falling into 95% CI in the visual predictive check. Plots of predicted versus observed concentrations, population/individual-weighted residuals (PWRES and IWRES) versus predictions/time, showed residuals uniformly distributed around the predictive values.

The final model was exported to Simulx 2020R1 in order to simulate 2000 individual time kill curves (1000 for CAMHB and milk, respectively), and then the *f*AUC/MIC range and the final bacterial count at 24 h for each simulated profile was recorded and used to conduct the I_max_ model, in order to determine the *f*AUC/MIC endpoints for reductions of −1, −2 and −3 log_10_CFU/mL for CAMHB and milk, respectively.

### 3.3. Determination of Marbofloxacin PK/PD Cutoff Values

An I_max_ model was conducted in order to determine the exposure-bacterial count difference (log_10_CFU/mL) between 0–24 h that allow to calculate the cutoff values of *f*AUC/MIC of MFX in CAMHB and goat milk.

**Figure 7:**
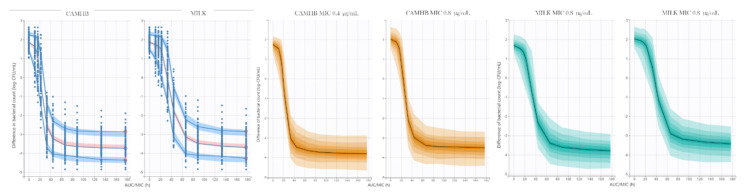
Visual predictive check of final Imax model of MFX against CNS (**left**); 95% CI of simulated Imax model of MFX in CAMHB for MIC values of 0.4 and 0.8 µg/mL (**middle**); 95% CI of simulated Imax model of MFX in milk of infected mammary gland for MIC values of 0.4 and 0.8 µg/mL (**right**).

The final model fitted well with the data, as can be seen in the visual predictive check, where most of the data falls within 95% confidence interval (Figure 7). Plots of predicted versus observed concentrations, population/individual-weighted residuals (PWRES and IWRES) versus predictions/time, showed residuals uniformly distributed around the predictive values.

Goat milk significantly increased IC_50_, and MIC increased E_0_ and IC_50_, respectively. Correlations between all model parameters were included because it significantly reduced the IIV and the likelihood ratio tests.

The final model was exported to Simulx 2020R1, and 2000 exposure-bacterial count difference curves (1000 for CAMHB and milk, respectively) were simulated (Figure 7), and *f*AUC/MIC values with 95% confidence interval were calculated for reductions of −1, −2 and −3 log_10_ CFU/mL reductions in bacterial count difference.

Generalized linear mixed-effects model analysis of PK/PD endpoints data showed a good fit, with AIC, BIC and −2LL values of −31727.45, −31685.62 and 15870.73, respectively. Culture medium (*p* ˂ 0.0001), MIC value (*p* ˂ 0.0001) and the value of reductions in bacterial count difference (*p* ˂ 0.0001), significantly affected the PK/PD cutoff values of *f*AUC/MIC for marbofloxacin. *f*AUC/MIC endpoints in goat milk were higher than CAMHB, higher for strains with MIC value of 0.8 µg/mL and higher accordingly to the magnitude of reductions in bacterial count difference, as could be seen in Table S1 of Supplementary Files.

### 3.4. PK/PD Analysis of Marbofloxacin against Coagulase Negative Staphylococci Isolated from Goat Mastitis

A PK/PD analysis of MFX against CNS was conducted by integrating 95% CI of MFX *f*AUC_first_/MIC and *f*AUC_mean_/MIC values obtained from goat serum and milk of infected mammary glands, and the MFX antimicrobial effect against CNS in CAMHB (considered to be equivalent to serum) and goat milk obtained from the I_max_ model.

**Figure 8:**
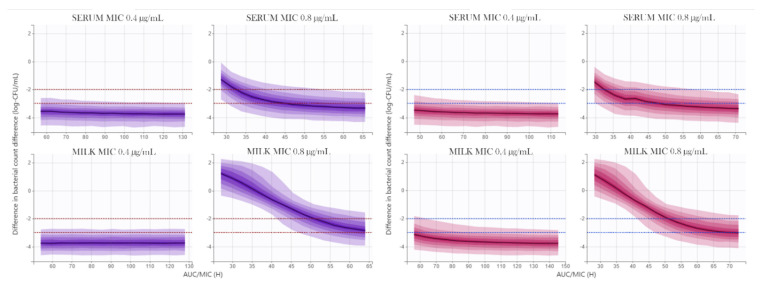
Bacterial count reductions of CNS (log_10_ CFU/mL difference) corresponding with 95% CI range of *f*AUC_first_/MIC, for serum and milk of infected mammary glands, discriminated by MIC value (**left**); Bacterial count reductions of CNS (log_10_ CFU/mL difference) corresponding with 95% CI range of *f*AUC_mean_/MIC, for serum and milk of infected mammary glands, discriminated by MIC value (**right**). Dashed horizontal lines correspond to reductions of −2 and −3 log_10_ CFU/mL.

For this purpose, a range of *f*AUC_first_/MIC and *f*AUC_mean_/MIC values corresponding with the 95% CI for serum and milk of infected udders, for MIC values of 0.4 and 0.8 µg/mL, were simulated with the MFX Imax model in Simulx 2020R1. The results are exposed in Figure 8. The predicted reductions in log_10_CFU/mL for 5% percentile, median and 95% percentile of predicted *f*AUC_first_/MIC and *f*AUC_mean_/MIC values of MFX, in serum (CAMHB) and milk of infected mammary glands are presented in Table S2 of Supplementary Files. In summary, serum and milk of the infected udder *f*AUC_first_/MIC values corresponding to 5% percentile, produced reductions of bacterial count > 2 log of CNS strains, and also when *f*AUC_mean_/MIC were used, taking into account the MIC_90_ value (0.4 µg/mL): −2.61 log_10_-CFU/mL for serum and −2.77 log_10_-CFU/mL for milk of the infected udder.

Finally, all animals finished the study with negative cultures in both mammary glands. Five animals showed negative bacterial cultures on the fourth day of treatment, while the other two animals did so on the fifth day. Moreover, both milk production and pH significantly changed during the MFX treatment. Milk production of both mammary glands increased over time (*p* = 0.0079), and was different between healthy and infected udders (*p* < 0.0001). Moreover, milk pH presented no difference during MFX treatment in healthy mammary glands (*p* = 0.257) and was 6.57 ± 0.03. In contrast, milk pH presented a significant reduction (*p* < 0.0001), and was 6.72 ± 0.04 at day 1 and 6.60 ± 0.07 at day 5.

## 4. Discussion

In this study, a multi-dose regimen of MFX, administered by intramuscular route at a dose of 10 mg/kg/24 h for five days, was evaluated in goats with mastitis produced by CNS. For this purpose, a PK/PD analysis was conducted with a nonlinear mixed-effect pharmacokinetic model in which serum and milk concentrations (from healthy and infected mammary glands) were included. Furthermore, pharmacokinetic predicted data was used to calculate the antimicrobial effect on CNS, by using a semi-mechanistic pharmacodynamic model that allow to calculate the bacterial count dynamics over time, in presence of different expositions to MFX. Moreover, clinical outputs were also included in the study: improvement in milk production, milk pH and microbiological cure, in order to compare PK/PD analysis outcomes with clinical outcomes.

### 4.1. Pharmacokinetic Modeling and Simulation

Most of pharmacokinetic data is obtained from healthy individuals, but antimicrobials are typically used in a sick patient. Many bacterial infections could produce pathophysiologic changes that may affect pharmacokinetic processes, such as absorption, distribution, metabolism and elimination, and hence, modify the pharmacokinetic behavior of the antimicrobial, leading to a different exposition to a microorganism. It could finally affect the efficacy of the antimicrobial [4]. This was the reason why we decided to conduct a multi-dose pharmacokinetic study with MFX in infected goats with mastitis produced by CNS. Since the PK study was at the same time an antimicrobial treatment, it allows us to obtain information about a clinical and microbiological cure. Moreover, not only serum, but milk MFX concentrations were measured, in order to determine the pharmacokinetic profile of MFX in the biophase, that could produce more accurate PK/PD indexes, and allow us to study the effect of infection on MFX passage from blood to milk.

The selected PK model used in this study was a modified two compartmental model, with two extra compartments included (corresponding to healthy and infected mammary glands), for which milk concentrations were measured. Moreover, an empty effect of each mammary gland was included in the model, since milking has an effect on antimicrobial elimination.

Pharmacokinetic analysis results showed that K_a_ and Q trend to decrease and Cl, Q_MH_ and VMH increased over time. Moreover, milk production of healthy mammary gland increased V1. These changes resulted in lower absorption and blood disposition of MFX. On the other hand, milk disposition of MFX was higher in infected, compared with healthy mammary glands. Inflammatory response produced by mammary gland infection could produce inflammatory mediators that are part of the called “acute phase response” and could affect some pharmacokinetic processes. K_a_ decrease could be related to hemodynamic changes in the site of administration, probably due to a local reaction produced by MFX injection. No local adverse reactions were observed in any animal after MFX administration, but a small irritation could be undetected, and still cause an increase in local blood flow. On the other hand, some mediators could increase muscle blood flow, and MFX absorption. Moreover, the observed decrease in Q and increase in Cl, Q_MH_ and VMH could be related with the systemic effects of inflammation on PK processes.

It is reported that inflammation and infection could lead to downregulation of drug-metabolizing enzymes and transporters (primarily of ABC superfamily), resulting in higher plasma concentrations and altering some distribution processes [31]. Many inflammatory mediators (interleukines, cytoquines, transforming growth factor, tumor necrosis factor or interferons) reduced the gene expression of cytochrome P450 complex in liver. On the other hand, inflammation and infection also downregulate the drug efflux transporters ATP-binding cassette (ABC) superfamily. These transporters are ubiquitous in the organism, and play a major role in drug transport of antimicrobials, among other drugs [31,32,33,34,35].

A specific ABC transporter, the “breast cancer resistance protein” (BCRP), and ABCG2 family transporter, is present in the human mammary gland and is responsible to excrete fluoroquinolones to milk [36]. BCRP was present in mammary glands of animals including sheep and goats, and it is reported that enrofloxacin and danofloxacin are substrates of BCRP [37,38,39,40,41,42]. Marbofloxacin could also be a substrate of BCRP, and inflammation and infection could lead to a downregulation of this transporter, leading to lower passage to milk in the first days of the study. This could explain the increase of Q_MH_ in days 4 and 5.

Furthermore, milk production increased in both healthy and infected mammary glands during MFX treatment, but was higher in healthy udders. In mammary gland infections, inflammatory mechanisms are generated and could have an effect on the uninfected gland, as described by other authors on goats [43,44]. In this way, subclinical infection in one breast medium could be reducing the production of the other healthy medium. The lower milk disposition of MFX in healthy mammary glands could be explained by the passage of MFX from the blood to goat milk, and the dilution effect that occurs when milk production increases, so that the MFX is diluted by a higher milk volume, according with a higher VMH.

In summary, downregulation of CYP, ABC transporters and increase in milk production could in part explain the context-sensitive pharmacokinetic behavior of MFX in serum and milk of goats with mastitis produced by CNS.

Another important aspect which could be affected by the abovementioned pharmacokinetic changes of MFX in goats with mastitis is the presence of residues in milk and the withdrawal period needed to warrantee that milk concentration of MFX in milk is below the recommended maximum residue limits (MRL). MFX milk concentration of healthy udders was above the LLOQ (0.025 µg/mL) in six animals until 36 h after the last administration, with a mean concentration of 0.091 ± 0.052 µg/mL. Moreover, in mastitic udders all animals presented concentrations above LLOQ at 36 h, with a mean concentration of 0.110 ± 0.065 µg/mL, and even one goat presented quantifiable concentrations until 48 h (0.099 µg/mL). These results indicate that mastitis could increase the permanence of MFX in goat milk, and this should be taken into account in order to estimate the withdrawal periods. Withdrawal period for MFX in bovine milk is 36 h for a dose regimen of 2 mg/kg/24 h and the MRL for bovine milk is 0.075 µg/mL [45,46]. Marbofloxacin is not approved for goats, and no withdrawal period is stablished for goat milk, and extrapolations from bovine are not possible, because previous results of Lorenzutti et al. (2017) [16] showed different pharmacokinetic behavior of MFX between goats and cows. Both improper withdrawal time (often due to extra-label use of drugs) and disease status of the animals are considered risk factors for the development of residues in food-producing animals [47]. Most withdrawal periods are determined from healthy animals and not in the diseased population in which the antimicrobial will be used, increasing the risk of residues presence. The determination of withdrawal periods for MFX in goat milk is needed, and the results of this study showed that the use of a mastitis model should be more correct in order to determine the real withdrawal time for a safer use of MFX for the treatment of goat mastitis.

### 4.2. Pharmacodynamic Modeling and Simulation

In order to characterize the exposition–effect relationship of MFX against CNS in MHB and goat milk, static time kill curves assays were carried out with thirteen CNS strains previously isolated from goat mastitis [16]. The objective to include this number of strains was to include bacterial pharmacodynamic variability in the semi-mechanistic model.

The final semi-mechanistic model consisted in bacterial net growth and MFX killing effect sub-models. The bacterial growth sub-model showed that both k_g_ and N_max_ were similar for MHB and goat milk, indicating that medium culture had no significant effects on the bacterial sub-model. On the other hand, the use of milk as a culture medium significantly increased EC_50_, showing that maximum effect of MFX could be reached against CNS, but with a higher concentration. It is important to note that the semi-mechanistic model was conducted with free-drug concentrations, so an increase in EC_50_ should not be produced by protein binding. Increased MIC values in milk compared to broth were previously reported for fluoroquinolones and other groups of antimicrobials with *Staphylococcus aureus* and *Escherichia coli* [48,49]. One explanation that the authors of both studies proposed is that some components of milk could bind antimicrobials, reducing its free concentration. Instead of protein binding, goat milk presents a high content of fat liposomes and somatic cells, which could represent a reservoir of MFX.

Additionally, the MIC value of the CNS strains increased EC_50_. MIC and EC_50_ are both potency parameters, but MIC is actually considered a hybrid pharmacodynamic parameter, since it is influenced by other pharmacodynamic and measurement conditions (the time that the measure is taken, the initial inoculum and the operator variability). In fact, the relationship of MIC and EC_50_ could be visualized by the following equation [7,49]:

$$\mathrm{MIC}={\;\mathrm{EC}}_{50}-{\left(\frac{k_{g}-0.29}{E_{\max}-\left(k_{g}-0.29\right)}\right)}^{\frac{1}{\gamma }}$$

In this manner, it could be seen that MIC and EC_50_ present a proportional relationship.

Finally, MIC influenced the initial inoculum N_0_. It could be related to inter-occasion variability in the initial inoculum of each experiment, and it has no biological meaning, but was conserved in the final model because it presented statistical significance, reduced the likelihood ratio tests and improved the predictive capability of the model.

### 4.3. Determination of Marbofloxacin PK/PD Cutoff Values

After pharmacodynamic simulation in Simulx 2020R1, 2000-time kill curves of MFX against CNS were obtained (1000 for each culture medium), and then, *f*AUC/MIC values and bacterial count differences between 24 h and time 0 were used to conduct and I_max_ model in order to predict *f*AUC/MIC endpoints related with reductions of −1, −2 and −3 log_10_CFU/mL. For CAMHB, −2 log_10_ reduction was achieved with a median *f*AUC/MIC (95% CI) of 26.66 (22.26–36.64) and 32.28 (26.57–48.35) for MIC values of 0.4 and 0.8 µg/mL, respectively; and −3 log_10_ reduction (bactericidal effect) with 32.26 (24.81–81.50) and 41.39 (29.38–128.01) for MIC values of 0.4 and 0.8 µg/mL, respectively. It is generally accepted for Gram-positive bacteria an endpoint of *f*AUC/MIC > 30–50 to achieve bactericidal or eradication effect [3,5,50,51,52]. Based on our results, higher *f*AUC/MIC values are needed in order to reach the PK/PD endpoint in 95% of the studied bacterial population, being similar to those reported for Gram-negative bacteria. For goat milk, −2 log_10_ reduction was achieved with a median *f*AUC/MIC (95% CI) of 41.48 (35.29–58.73) and 51.91 (39.09–131.63) for MIC values of 0.4 and 0.8 µg/mL, respectively; and −3-log reduction (bactericidal effect) with 51.04 (41.6–82.1) and 65.65 (46.68–210.16) for MIC values of 0.4 and 0.8 µg/mL, respectively. These endpoints are significantly higher than CAMHB, and *f*AUC/MIC values corresponding with 95% CI, could be related with toxic effects.

These *f*AUC/MIC endpoints were obtained from static in vitro experiments, and the immune and granulocyte-mediated killing effect that occurs in an immunocompetent individual was not considered. Some in vivo studies evaluated this effect in murine pneumonia and tight infection models, and reported that granulocyte-mediated bacterial clearance is a saturable mechanism, and half saturation occurs at inoculums of ≈10^6^ CFU/g of tissue. Moreover, the maximal granulocyte kill effect is ≈1–2 log_10_ CFU/g. Authors suggest that an antimicrobial-killing effect of at least 2 log_10_CFU/g in the first 24 h should be adequate to avoid granulocyte-killing saturation and improve the outcome [53,54,55]. Taking it into account, it could be possible to consider a −2 log reduction in bacterial count for immunocompetent patients, as long as the antimicrobial excerpt an initial reduction of at least 2 log_10_CFU in the first 24 h of the initiation of therapy. Marbofloxacin, as well as other fluoroquinolones, present a rapid killing phase after initiation of therapy, and this could be an advantage for treatment success.

### 4.4. PK/PD Analysis of Marbofloxacin against Coagulase Negative Staphylococci Isolated from Goat Mastitis

The PKPD analysis of the proposed multi-dose regimen of MFX for the treatment of goat mastitis produced by CNS was conducted with Simulx 2020R1. *f*AUC_first_/MIC and *f*AUC_mean_/MIC 95% CI values for serum and milk of the infected mammary gland, obtained from the pharmacokinetic model, were used to calculate the 95% CI bacterial count difference. As shown in Table S2 of Supplementary Files and Figure 8, MFX efficacy was higher in the first day of treatment, because presented higher *f*AUC/MIC values. As we discussed in pharmacokinetic analysis, it could be due to the effect of inflammation and infection on downregulation of CYP-450, ABC transporters and milk production. In this case, these factors could play a beneficial role in MFX therapy, allowing to excerpt a fast bacterial count reduction in the early phase of treatment, and minimizing the risk of saturation of granulocyte-killing capability. Moreover, efficacy criteria did not meet for either serum or milk of the infected mammary gland when the higher MIC value was considered (0.8 µg/mL).

These results are in agreement with the prediction of previous PK/PD analysis by Monte Carlo simulation, which proposed an optimal MFX dose of 10 mg/kg/24 h by intramuscular route [16], and with the clinical outcomes, which showed microbiological and clinical cure in all goats between the fourth and fifth day. It is important to highlight that only a low initial bacterial inoculum (approximately 10^6^ CFU/mL) was used to time kill curve experiments, and higher inoculum could increase EC_50_, decrease E_max_ or both, resulting in higher *f*AUC/MIC cutoff values for a determined bacterial count reduction endpoint [56,57,58,59]. It could be relevant in some cases of severe clinical mastitis, in which bacterial burden could be higher than 10^6^–10^7^ CFU/mL.

## 5. Conclusions

According to these results, it could be concluded that the proposed dose regimen of MFX (10 mg/kg/24 h) administered by intramuscular route for five days was adequate for the treatment of goat mastitis produced by CNS, resulting in microbiological and clinical cure of all animals. Anyway, it is important to highlight that MFX should not be the first choice for the treatment of goat mastitis, taking into account that fluoroquinolones are considered critically important antimicrobial agents. The use of fluoroquinolones in food producing animals vary among different world areas. For example, this group is prohibited in the United States (for food producing animals), but not in Europe or South America. Moreover, an extra-label use of MFX is done in goats, since it is not approved for this species. This is why PK/PD studies in goats are needed.

Another important conclusion of this study is that the use of an animal model with infection is highly recommended, since it provide important pharmacokinetic information about the effect of the infection on the pharmacokinetic behavior of the antimicrobial, that cannot be obtained from healthy animal models. MFX showed higher disposition in goat milk in the early phase of the treatment, that could be explained by downregulation of CYP, ABC transporters and lower milk production. Then, MFX presented a faster elimination from serum and milk at the end of the study. The context-sensitive pharmacokinetics reported in this study would not be observed if only a single-dose study was performed, and this provide evidence of the importance to replicate in PK/PD studies the same conditions of disease and posology present in the clinical setting. These factors could lead to significant changes in the pharmacokinetic behavior of the antimicrobial over time, and this have direct impact on drug disposition in the biophase, and finally in antimicrobial efficacy.

One limitation of the animal model used in this study is that marbofloxacin (and other fluoroquinolones) are principally used (extra-label use) for treat clinical mastitis in goats, and not for subclinical mastitis. Some differences between subclinical and clinical mastitis in goats, regarding to milk production, inflammation or tissue damage of the udders could result in different alterations of the pharmacokinetic behavior of MFX, and could lead to different results. The authors decided to use animals with subclinical mastitis because animal welfare reasons. More precisely, enrolling animals with clinical mastitis, would produce a delay in the beginning of the treatment, because this study was carried out with animals from different herds located out of our university, in private productions, and one of the inclusion criteria was the isolation of the pathogen, as a part of mastitis diagnosis, and some days were needed until culture results were available. Since the evolution of clinical mastitis in goats is relatively fast, animals need to be treated as soon as possible, in order to warrantee animal welfare. In this way, it was considered that the use of an animal model of subclinical mastitis should satisfy the objective of this study of include animals with disease and this model should be more representative and realistic that a pharmacokinetic study of an antimicrobial in healthy animals, where nor infection or inflammation are present.

On the other hand, pharmacodynamic modeling showed that *f*AUC/MIC cutoff values for MFX against CNS are context-sensitive, and was higher in goat milk compared with CAMHB, and therefore, use of adequate PK/PD cutoff values for each biophase is highly recommended in order to conduct more precise PK/PD analysis. Moreover, the agreement of PK/PD analysis and clinical outcomes showed the importance of pharmacokinetic and pharmacodynamic modeling as a powerful tool for the evaluation of antimicrobial efficacy and dose optimization.

## Figures and Tables

**Table 1:** Milk production, somatic cell count and pH of healthy and infected mammary glands of goats included in the study.

Parameter	Healthy Gland	Infected Gland	*p*-Value
Milk production (L/day)	0.26 ± 0.15	0.12 ± 0.15	0.045
SCC (×10^3^)/mL	541 ± 126	1389 ± 173	0.0002
pH	6.57 ± 0.03	6.72 ± 0.04	0.0001

Data are presented as mean ± SD. Significance value *p* < 0.05; SCC: Somatic cell count.

**Table 2:** Final model parameters of the multi-dose regimen of marbofloxacin (10 mg/kg/24 h) administered by intramuscular route for five days in lactating goats with mastitis produced by CNS (*n* = 7).

	Estimates (RSE; %)	IIV (RSE; %)	IOV (RSE; %)	Shrinkage (%)
Parameter estimates				
K_apop_ (h^−1^)	10.8 (14.9)	0.253 (34.2)	0.24 (18.8)	44.8
Cl_pop_ (mL·Kg^−1^·h^−1^)	0.237 (10.1)	0.235 (27.8)	0.0758 (25)	−0.19
V1_pop_ (L·Kg^−1^)	0.953 (22.3)	0.547 (26.9)	0.071 (18.4)	45.2
Q_pop_ (mL·Kg^−1^·h^−1^)	0.0412 (28.3)	0.1 (94.7)	0.182 (37.4)	9.89
V2_pop_ (L·Kg^−1^)	1.53 (20.1)	0.526 (27.4)	0.151 (17.7)	−0.743
Q_MHpop_ (mL·Kg^−1^·h^−1^)	0.0239 (31.8)	0.498 (30.1)	0.33 (18)	15
VMH_pop_ (L·Kg^−1^)	0.0436 (32)	0.569 (27.2)	0.0256 (243)	24.7
Q_MIpop_ (mL·Kg^−1^·h^−1^)	0.0211 (19.8)	0.494 (30.1)	0.318 (17.5)	−6.02
VMI_pop_ (L·Kg^−1^)	0.05 (17)	0.437 (28.1)	0.0215 (833)	13.7
Covariates estimates				
beta_k_a__DAY_2	1.45 (12.1)	-	-	-
beta_k_a__DAY_3	−0.224 (69.4)	-	-	-
beta_k_a__DAY_4	−1.21 (13.1)	-	-	-
beta_k_a__DAY_5	−0.44 (34.5)	-	-	-
beta_Cl_DAY_2	−0.117 (58.6)	-	-	-
beta_Cl_DAY_3	0.0702 (91.7)	-	-	-
beta_Cl_DAY_4	0.166 (37.6)	-	-	-
beta_Cl_DAY_5	0.29 (20.4)	-	-	-
beta_V1_VOL_HEALTHY	0.543 (36.9)	-	-	-
beta_Q_DAY_2	−0.976 (32)	-	-	-
beta_Q_DAY_3	−1.01 (31.3)	-	-	-
beta_Q_DAY_4	−0.561 (54.3)	-	-	-
beta_Q_DAY_5	−0.467(63.8)	-	-	-
beta_Q_MH__DAY_2	0.306(104)	-	-	-
beta_Q_MH__DAY_3	−0.409(70.8)	-	-	-
beta_Q_MH__DAY_4	0.781(39.1)	-	-	-
beta_Q_MH__DAY_5	0.864(33.7)	-	-	-
beta_VMH_DAY_2	0.102(283)	-	-	-
beta_VMH_DAY_3	0.311(79.1)	-	-	-
beta_VMH_DAY_4	0.84(29.8)	-	-	-
beta_VMH_DAY_5	1.15(21.3)	-	-	-
Error model parameters				
b2	0.253(0.00763)	-	-	-
b3	0.233(0.0745)	-	-	-
b4	0.201(0.043)	-	-	-

RSE: relative standard error; IIV: inter-individual variability; IOV: inter-occasion variability.

**Table 3:** Descriptive statistics of simulated *f*AUC_first_/MIC and *f*AUC_mean_/MIC values of an intramuscular multi-dose regimen of marbofloxacin (10 mg/kg/24 h) for five days in goats with mastitis produced by CNS.

Variable	Median	Min	Max	P(05)	P(95)
Serum					
*f*AUC_first_/MIC (MIC = 0.4 µg/mL)	86.32	37.51	201.22	57.08	131.24
*f*AUC_first_/MIC (MIC = 0.8 µg/mL)	43.16	18.76	100.61	28.54	65.62
*f*AUC_mean_/MIC (MIC = 0.4 µg/mL)	72.68	31.64	185.88	47.45	113.45
*f*AUC_mean_/MIC (MIC = 0.8 µg/mL)	45.42	19.78	116.18	29.66	70.9
Milk (infected mammary gland)					
*f*AUC_first_/MIC (MIC = 0.4 µg/mL)	83.74	14.84	200.57	54.10	127.99
*f*AUC_first_/MIC (MIC = 0.8 µg/mL)	41.87	7.42	100.29	27.05	64.00
*f*AUC_mean_/MIC (MIC = 0.4 µg/mL)	89.87	41.89	223.92	56.42	145.55
*f*AUC_mean_/MIC (MIC = 0.8 µg/mL)	44.93	20.95	111.96	28.21	72.78

**Table 4:** Final model parameters of marbofloxacin time kill curves against CNS isolated from goat mastitis.

	Estimates (RSE; %)	IIV (RSE; %)	Shrinkage (%)
Parameter estimates			
N_maxpop_ (log_10_ CFU/mL)	7.51 (0.897)	0.0297 (38.3)	11.3
k_gpop_ (h^−1^)	0.351 (7.65)	0.533 (14.7)	−1.72
N_0pop_ (log_10_ CFU/mL)	6 (2.02)	0.0201 (106)	4.72
gamma_pop_	3.78 (13.2)	0.715 (14.8)	3.38
K_maxpop_ (h^−1^)	0.283 (6.74)	0.515 (12.4)	−1.12
EC_50pop_ (µg/mL)	0.165 (8.76)	0.0842 (34.8)	−8.52
Covariate estimates			
beta_N_0__MIC	−0.129 (28.7)	-	-
beta_EC_50__MILK	0.453 (11.1)	-	-
beta_EC_50__MIC	2.13 (8.12)	-	-
Correlations estimates			
Corr_k_g__K_max_	0.988 (1.26)		-
Error model parameters			
a	0.512 (3.37)	-	-

RSE: relative standard error; IIV: inter-individual variability.

## Data Availability

The data presented in this study are available on request from corresponding author.

## Supplementary Materials

The following are available online at https://www.mdpi.com/article/10.3390/ani11113098/s1, Table S1. Final Imax model parameters of marbofloxacin in CAMHB and goat milk against coagulase-negative staphylococci isolated from goat mastitis, Table S2. Results of PK/PD analysis in serum and milk of the proposed dose regimen of marbofloxacin (10 mg/kg/24h) administered by intramuscular route in goats with mastitis produced by coagulase-negative staphylococci.
