# Supplementary material for: PK/PD Analysis by Nonlinear Mixed-Effects Modeling of a Marbofloxacin Dose Regimen for Treatment of Goat Mastitis Produced by Coagulase-Negative Staphylococci
Source: Animals (Basel). 2021 Oct 29;11(11):3098. doi: 10.3390/ani11113098 (PMC8614466; doi:10.3390/ani11113098)
Supplement: Supplementary file 1 [file animals-11-03098-s001.zip › animals-1350371-supplementary.pdf]

Supplementary Materials

PK/PD Analysis by Nonlinear Mixed-Effects Modeling of a Marbofloxacin Dose Regimen for Treatment of Goat Mastitis Produced by Coagulase-Negative Staphylococci

Augusto Matías Lorenzutti [1], Juan Pablo Vico [1], Juan Manuel Serrano-Rodríguez [2,*], Martín Alejandro Himelfarb [1], Manuel Ignacio San Andrés-Larrea [3], José Julio de Lucas-Burneo [3] and Nicolás Javier Litterio [1]

[1] Facultad de Ciencias Agropecuarias, IRNASUS CONICET-Universidad Católica de Córdoba, Armada Argentina 3555 (X5016DHK), Córdoba, Argentina; matiaslorenzutti@ucc.edu.ar (A.M.L.), juanpablo.vico@ucc.edu.ar (J.P.V.);
martinhimelfarb@ucc.edu.ar (M.A.H.); nlitterio@ucc.edu.ar (N.J.L.)

[2] Department of Nursing, Pharmacology and Physiotherapy, Pharmacology Area, Faculty of Veterinary Medicine, Universidad de Córdoba, Córdoba, Spain

[3] Department of Pharmacology and toxicology, Faculty of Veterinary Medicine, Universidad Complutense de Madrid, Madrid, Spain; misanand@vet.ucm.es (M.I.S.A.-L.); delucas@vet.ucm.es (J.J.d.L.-B.)

* Correspondence: q22seroj@uco.es

Table S1. Final Imax model parameters of marbofloxacin in CAMHB and goat milk against coagulase-negative staphylococci isolated from goat mastitis.

	**Estimates (RSE;%)**	**IIV (RSE;%)**	**Shrinkage (%)**
Parameter estimates			
E_{0pop}	1.46 (3.27)	0.16 (5.02)	−0.105
$gamma_{pop}$	4.59 (5.35)	0.756 (5.01)	0.383
I_{maxpop}	5.59 (0.701)	0.0988 (5.03)	−0.692
IC_{50pop}	19.4 (1.98)	0.087 (5.06)	−0.37
Covariate estimates			
beta_E_0_MIC	0.391 (13)	-	-
beta_IC_{50}_ MILK	0.451 (2.62)	-	-
beta_IC_{50}_MIC	0.386 (7.7)	-	-
Correlations estimates			
corr_IC_{50}_E_0	0.163 (42.6)	-	-
corr_I_{max}_E_0	0.401 (14.9)	-	-
corr_gamma_E_0	0.0905 (77.7)	-	-
corr_I_{max}_IC_{50}	0.0807 (87.9)	-	-
corr_gamma_IC_{50}	0.255 (26.2)	-	-
corr_gamma_I_{max}	-0.186 (36.8)	-	-
Error model parameters			
b	0.0147 (2.12)	-	-

RSE: Relative standard error; IIV: Inter-individual variability.

Table S2. Results of PK/PD analysis in serum and milk of the proposed dose regimen of marbofloxacin (10 mg/kg/24h) administered by intramuscular route in goats with mastitis produced by coagulase-negative staphylococci.

AUC value	AUC/MIC	Median	P05	P95
Serum (MIC = 0.4 μg/mL)				
AUC_{first}	P05 = 57.08	−3.55436	−4.56796	−2.60947
	Median = 88.88	−3.70868	−4.68305	−2.85198
	P95 = 131.24	−3.75305	−4.66661	−2.94904
AUC_{mean}	P05 = 47.45	−3.46896	−4.50259	−2.39889
	Median = 75.734	−3.68008	−4.66156	−2.81225
	P95 = 113.448	−3.74024	−4.65319	−2.96586
Serum (MIC = 0.8 μg/mL)				
AUC_{first}	P05 = 28.64	−1.27798	−2.70029	−0.0700124
	Median = 44.48	−2.95776	−4.09258	−1.72183
	P95 = 65.61	−3.32412	−4.31541	−2.22557
AUC_{mean}	P05 = 29.66	−1.45019	−2.87012	−0.305033
	Median = 47.336	−2.96934	−3.99165	−1.75763
	P95 = 70.9	−3.2893	−4.23362	−2.37383
Milk of infected mammary gland (MIC = 0.4 μg/mL)				
AUC_{first}	P05 = 54.1	−3.7477	−4.61082	−2.77467
	Median = 85.78	−3.74086	−4.61906	−2.78744
	P95 = 127.99	−3.739	−4.59428	−2.73958
AUC_{mean}	P05 = 56.42	−3.16079	−4.27621	−1.9656
	Median = 94.64	−3.61244	−4.57233	−2.64988
	P95 = 145.55	−3.70789	−4.6152	−2.89966
Milk of infected mammary gland (MIC = 0.8 μg/mL)				
AUC_{first}	P05 = 27.05	1.22958	−0.344938	2.27413
	Median = 42.89	−1.00153	−2.38362	0.632599
	P95 = 64	−2.85098	−3.93108	−1.55831
AUC_{mean}	P05 = 28.21	1.23185	−0.488319	2.34401
	Median = 47.29	−1.53703	−2.9647	−0.45877
	P95 = 72.755	−2.98746	−4.01723	−1.79676

P05: 5% percentile; P95: 95% percentile. Results are expressed in reduction of log_{10} CFU/mL.
